# Implementation fidelity of a clinical medication review intervention: process evaluation

**DOI:** 10.1007/s11096-018-0615-y

**Published:** 2018-03-20

**Authors:** F. Willeboordse, F. G. Schellevis, M. C. Meulendijk, J. G. Hugtenburg, P. J. M. Elders

**Affiliations:** 1 Department of General Practice & Elderly Care Medicine, Amsterdam Public Health Research Institute, VU University Medical Center, Amsterdam, The Netherlands; 2 NIVEL, Netherlands Institute for Health Services Research, Utrecht, The Netherlands; 3 Department of Information and Computing Sciences, Utrecht University, Utrecht, The Netherlands; 4 Department of Public Health and Primary Care, Leiden University Medical Center, Leiden, The Netherlands; 5 Department of Clinical Pharmacology & Pharmacy, VU University Medical Center, Amsterdam, The Netherlands

**Keywords:** Drug-related problems, Implementation barriers, Implementation fidelity, Medication review, Process evaluation

## Abstract

*Background* Implementation of clinical medication reviews in daily practice is scarcely evaluated. The Opti-Med intervention applied a structured approach with external expert teams (pharmacist and physician) to conduct medication reviews. The intervention was effective with respect to resolving drug related problems, but did not improve quality of life. *Objective* The objective of this process evaluation was to gain more insight into the implementation fidelity of the intervention. *Setting* Process evaluation alongside a cluster randomized trial in 22 general practices and 518 patients of 65 years and over. *Method* A mixed methods design using quantitative and qualitative data and the conceptual framework for implementation fidelity was used. Implementation fidelity is defined as the degree to which the various components of an intervention are delivered as intended. *Main outcome measure* Implementation fidelity for key components of the Opti-Med intervention. *Results* Patient selection and preparation of the medication analyses were carried out as planned, although mostly by the Opti-Med researchers instead of practice nurses. Medication analyses by expert teams were performed as planned, as well as patient consultations and patient involvement. 48% of the proposed changes in the medication regime were implemented. Cooperation between expert teams members and the use of an online decision-support medication evaluation facilitated implementation. Barriers for implementation were time constraints in daily practice, software difficulties with patient selection and incompleteness of medical files. The degree of embedding of the intervention was found to influence implementation fidelity. The total time investment for healthcare professionals was 94 min per patient. *Conclusion* Overall, the implementation fidelity was moderate to high for all key components of the Opti-Med intervention. The absence of its effectiveness with respect to quality of life could not be explained by insufficient implementation fidelity.

**Electronic supplementary material:**

The online version of this article (10.1007/s11096-018-0615-y) contains supplementary material, which is available to authorized users.

## Impact on practice

- Performing medication analyses for clinical medication reviews by external expert teams is feasible.
- Cooperation between fixed expert teams, consisting of a physician and a pharmacist and the use of an online decision-support medication evaluation facilitates the implementation of clinical medication reviews.
- Time, cost reimbursement, training and a dedicated practice nurse or coordinator in the GP practice seem to be necessary for successfully implementing clinical medication reviews. In addition, software programs for patient selection, exchange of medical and medication files and outcomes of medication evaluation are needed.

## Introduction

Implementation fidelity is defined as the degree to which the various components of an intervention are delivered as intended [1]. Convenience of use and degree of implementation exert considerable influence on the applicability of a complex healthcare intervention in daily practice. Implementation fidelity gives researchers and practitioners a better understanding of how and why an intervention is effective or ineffective, and the extent to which health outcomes can be improved. Implementation fidelity reflects the adherence to content, frequency, duration and coverage of the intervention. In addition, there may be moderating factors that influence the degree of implementation fidelity [1, 2]. As long as the evaluation of the implementation fidelity has not been performed, it remains unclear whether ineffectiveness is due to a poor implementation of the intervention or inadequacies inherent to the intervention itself.

In this study, the complex intervention of a clinical medication review (CMR) has been evaluated. A CMR is a structured, critical examination of the patient’s medicines with the objective of reaching an agreement with the patient about treatment, optimising the impact of medicines, minimising the number of drug related problems (DRPs) and reducing waste [3]. CMRs can improve the appropriateness of drug prescribing and medication use and are increasingly used and recommended in primary care [4–7]. However, in daily practice the implementation of CMRs is difficult and time consuming. [8, 9] A recent review highlights the need for research on intervention development and process evaluations to improve the understanding of how effective interventions to prevent potentially inappropriate prescribing can be sustained and ultimately be translated into improvements in patient outcomes [10]. Therefore, the Opti-Med randomised controlled trial (RCT) was recently carried out in a primary care population to test the effectiveness of CMRs on the quality of life and DRPs.

**Fig. 1:**
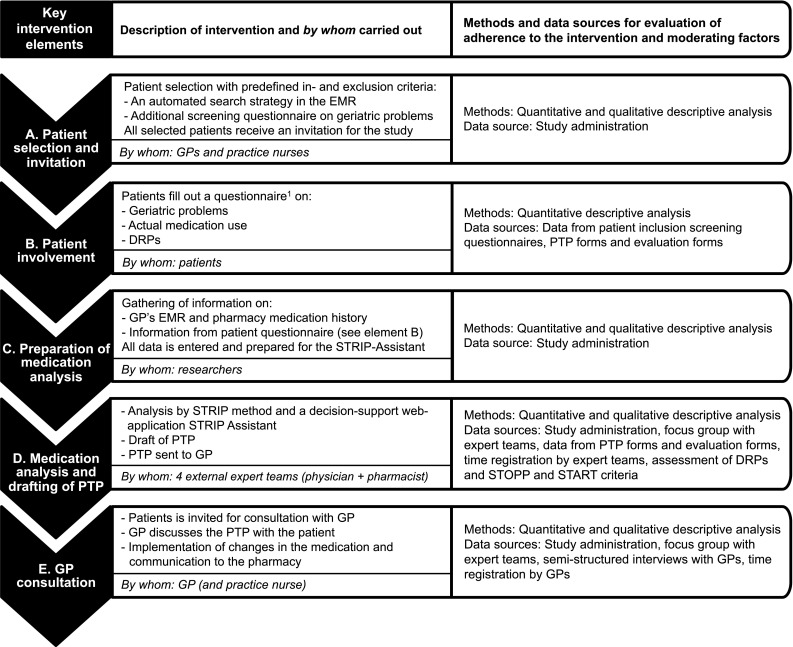
Overview of the Opti-Med intervention and important elements for the process evaluation. *DRPs* drug related problems, *EMR* electronic medical record, *GP* general practitioner, *PTP* pharmacotherapeutic treatment plan, *START* screening tool to alert doctors to right treatment, *STOPP* screening tool of older person’s prescriptions, *STRIP* systematic tool to reduce inappropriate prescribing, *STRIPA* systematic tool to reduce inappropriate prescribing assistant. ^1^Questionnaire by Willeboordse et al. [19]

The Opti-Med study design and its results have been published separately [11, 12]. In short, The Opti-Med study was designed as a cluster RCT in 22 general practices (Fig. 1) [11]. We studied the effects of CMRs on quality of life and DRPs in 518 older patients (≥ 65 year). Patients were selected and invited when they chronically used one or more prescribed drugs and newly presented themselves to the general practitioner (GP) with one or more geriatric problems (immobility, instability, incontinence and impaired cognition). Patient selection was facilitated by software specifically developed for the Opti-Med study based on electronic medical records (EMRs). CMRs were conducted by the expert teams according to a structured program using the STRIPA tool [13]. Patients in control practices received usual GP care with no specific attention to their medication use.

The Opti-Med study included three innovative CMR elements. First, medication analyses were carried out by trained external expert teams consisting of a pharmacist and a physician, not being the patient’s own GP and pharmacist.

The second innovative element was a new target group. We included patients of 65 years and over who chronically used ≥ 1 prescribed drug and had one or more geriatric problems, also called geriatric giants (immobility, instability, incontinence and impaired cognition) instead of polypharmacy patients, which is the usual target group. Inappropriate medication use may be associated with a higher risk on the occurrence and persistence of these geriatric problems. The nature of this association is complex, as the causes of these problems are multifactorial; however these geriatric problems are among the most common adverse drug reactions [14–19].

The third innovative element was the method of patient involvement. Patients gave input for the medication analyses by means of completing a questionnaire and discussed the results of the analyses during a consultation with their GP.

We hypothesized that these three elements would facilitate the implementation of CMRs in daily practice and thereby increase their effectiveness. The results of our effectiveness study showed that the Opti-Med CMRs indeed improved appropriate prescribing, i.e. more DRPs were identified and solved after 6 months of follow-up compared to usual GP care, but there was no effect on patients’ quality of life [12]. A process evaluation of the Opti-Med intervention could clarify whether the limited impact of the Opti-Med intervention was due to a poor implementation or due to inadequacies inherent to the intervention itself.

## Aim of the study

The aim of this process evaluation study is to gain more insight into the implementation fidelity of the Opti-Med CMR intervention in daily practice.

## Method

### Study design

This process evaluation was conducted alongside the Opti-Med RCT. Within the present study, the implementation fidelity of the Opti-Med intervention was evaluated. Quantitative data was collected from the start of the study and qualitative data was collected at the end of the study. For the evaluation we distinguished five key intervention components:

A. Patient selection and invitation by GPs and practice nurses to participate using EMRs through a newly developed software;
B. Patient involvement through a patient questionnaire [20];
C. Preparation of the medication analysis by practice nurses and Opti-Med researchers;
D. Medication analysis and drafting of a Pharmacotherapeutic Treatment Plan (PTP) by an expert team. The expert teams followed accredited online courses for CMRs and two face-to-face CMR workshops. An electronic medication evaluation tool, the Systematic Tool to Reduce Inappropriate Prescribing Assistant (STRIPA) [13] was used for the medication analysis;
E. GP consultation with the patient and implementation of the PTP.

## Conceptual framework for implementation fidelity

**Fig. 2:**
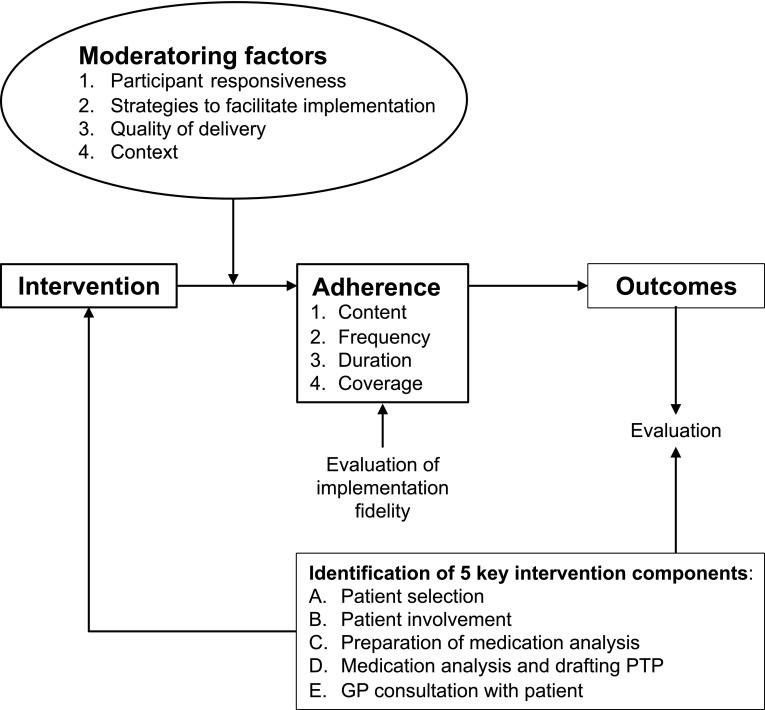
Adapted conceptual framework for implementation fidelity for the Opti-Med process evaluation. The measurement of implementation fidelity is the measurement of adherence of the categories content, frequency, duration and coverage

The adapted Conceptual Framework for Implementation Fidelity was used (Fig. 2) [1, 2]. The framework allows to evaluate both adherence to the intervention and to assess moderating factors for adherence to the intervention.

Adherence to the intervention includes the dimensions content, frequency, duration and coverage.

Moderating factors for adherence to the intervention include the dimensions participant responsiveness, strategies to facilitate implementation, quality of delivery and context.

Specific research questions and outcomes per key intervention component (A–E) for each dimension of the conceptual framework are presented in Tables 1 and 2. A subjective rating was used to evaluate the implementation fidelity and the researchers assigned the ratings for each dimension of the framework using four categories: very low, low, moderate, high. ‘Very low’ means that almost none of the intervention elements were carried out as planned, ‘low’ means that some elements have been carried out as planned, ‘moderate’ means that the majority of the elements have been carried out as planned and ‘high’ means that almost all elements have been carried out as planned.

**Table 1:** Research questions for the evaluation of adherence, data sources and outcomes for the implementation fidelity of the Opti-Med intervention

Key intervention components	Data source^1^	Specific research questions	Outcomes	Rating*
1. Evaluation of adherence: content				
1a. Patient selection	I	To what extent was the patient selection implemented as planned?	Patient selection was carried out as planned according to the inclusion criteria. However, in practice it was not fully carried out by practice nurses but researchers provided extensive support or carried it out completely	Moderate
1b. Patient involvement	IV	To what extent did the patient questionnaire information influence and tailor the PTP?	Patient questionnaire information was often used to tailor the PTP. Face-to-face patient contact might have resulted in more useful information according to the expert teams, e.g. compliance problems	High
1c. Preparation of medication analysis	I	To what extent was the preparation of the medication analyses implemented as planned?	The preparation of the medication analyses was carried out by the researchers, therefore not fully implemented as planned. The gathering of information (medical EMR data and medication data from pharmacy) was planned to be carried out by the practice nurses. Medication analysis preparation was deemed sufficient by the expert teams	Moderate
1d. Medication analysis	II	To what extent was the medication analysis implemented as planned? (structure, cooperation, STRIPA, knowledge and drafting the PTP)	Medication analysis by the expert team was carried out in a structured manner due to the use of the IT application STRIPA. Cooperation was good and complementary knowledge helpful. All expert teams formed fixed couples which improved cooperation and efficiency. Frequency, often once per month, also improved cooperation, efficiency and knowledge. All expert teams used primary care guidelines and applied STOPP and START criteria. The drafting of the PTP was deemed easy due to the structured STRIPA format but the lay-out and overview could be improved	High
1e. GP consultation	II, III	To what extent were patient consultations delivered and prepared as planned?	GPs differently performed the consultation: most GPs planned double consultation time and used a few minutes to prepare the consultations using the PTP form. In one practice, consultations were thoroughly prepared and discussed by phone, in another practice over half of the patients were visited at home. In two practices, the practice nurse did the consultation with the patient and only discussed major changes with the GP. As the result there was more attention for patient knowledge, compliance and preferences According to GPs the use of external expert teams brought advantages such as efficiency and feasibility (as needed when conducting CMRs for larger numbers of patients), objectivity, expertise, and extra convincing power towards the patient. However, they also considered a final evaluation by the GP always necessary but requiring a certain time-investment. The expert teams mentioned advantages like objectivity. Not knowing the patient may also circumvent preconceptions by the patients’ GP	High
2. Evaluation of adherence: frequency				
2a. Patient selection	I	How many times a patient selection was performed?	Patients were selected approximately every 2–3 months and a list with eligible patients was composed. Out of 112 possible lists, 105 (94%) lists were successfully processed. In total 3 lists could not be produced due to software problems and 4 lists were produced but not processed by the GP due to time constraints	High
2b. Patient involvement	IV, VI	How many patient questionnaires were completed and completed by the patient themselves? What was the influence of the patient input on the identified DRPs?	All questionnaires were filled in by the participants. 17% of the patients did not fill out the questionnaire independently but were assisted by family or other informal carers or visited at home by the researchers 19% of all DRPs were identified on the basis of patient questionnaire specific data on actual medication use, DRPs, geriatric problems and pain	High
2c. Preparation of medication analysis	I	How many CMRs were prepared?	All 518 CMRs were prepared as planned. For 11 patients the medication list from the community pharmacy was not received (in time), and the medication list provided by the patient and/or the GP was used	High
2d. Medication analysis	I, IV, V	How many medication analyses were performed? How many proposed interventions and DRPs were formulated? To what extent were the proposed interventions implemented as planned? Were there differences in implementation rate for different type of proposed interventions?	A medication analysis was performed for 274 of 275 participants in the intervention group (one drop-out before expert team started) and for all 243 control patients See Fig. 3 for the frequency, nature and implementation rate of the proposed interventions and drug related problems, including reasons for not follow-up the interventions. For 275 intervention patients, 1282 interventions were proposed by the external expert teams and documented on the pharmacotherapeutic treatment plans. Retrospectively, the researchers identified 1212 drug related problems with the DOCUMENT tool, out of these proposed interventions. In total, there were 8 patients without any DRPs The implementation rate was higher for non-pharmacological interventions than pharmacological interventions, 69.2% compared to 42.6% (*t* test *p* < 0.001) The implementation rate for addition of drug was higher than for cessation of drug, 46.7% compared to 34.7% (*t* test, *p* = 0.002)	High
2e. GP consultation	I	How many GP consultations were performed?	90% (247) of the PTPs were discussed with the patient by the GP	High
3. Evaluation of adherence: duration				
3a. Patient selection	I	What was the estimated duration to select a patient?	About 1 min per patient	NA
3b. Patient involvement	–	NA	NA	
3c. Preparation of medication analysis	I	What was the estimated duration to prepare a medication analysis? How many days were there between inclusion and GP consultation date?	15 min per patient (including gathering of information, enter data and process the PTP) Median (IQR) number of days between inclusion and the consultation was 33.0 (15–51) days 42% of the patients had their consultation within the planned 1 month after inclusion	NA
3d. Medication analysis	VII	What was the mean duration of a medication analysis by the expert team?	Mean [sd] 22 [17] minutes per expert team member per patient	NA
3e. GP consultation	VII	What was the mean duration of a GP consultation?	Mean [sd] 34 [19] minutes per patient	NA
4. Evaluation of adherence: coverage				
4a. General	I, VIII	What proportion of the selected patients was invited to participate? What proportion of the invited patients participated and how was the drop-out and follow-up? Were there differences between GP practices? Were there differences in patient characteristics between the responders and non-responders?	2401 patients were initially selected on the basis of their GP EMR, 2037 (85%) patients were invited to participate. 364 (15%) patients were excluded after selection by the GP because they were terminally ill or due to a specific reason why it was not desirable to invite the patient (range 4–33% between GP practices) 25% were included (range 12–33% between GP practices) 15% was considered not eligible (range 0–22% between GP practices) 41% did not respond at all (range 26–64% between GP practices) 19% declined to participate (range 8–31% between GP practices) See figure for the patient flow in the Opti-Med study in “Online resource 1” Patients who declined to participate did not differ in age as compared to participants, but among them there were significantly less women (χ^2^, *p* = 0.02) and they used less medication (*t* test, *p* < 0.001) Patients who did not respond did not differ in age and gender as compared to participants	High

*CMR* clinical medication review, *DRP* drug related problem, *EMR* electronic medical record, *GP* general practitioner, *IT* information technology, *NA* not applicable, *PTP* pharmacotherapeutic treatment plan, *START* screening tool to alert doctors to right treatment, *STOPP* screening tool of older person’s prescriptions, *STRIPA* systematic tool to reduce inappropriate prescribing assistant

^1^I. Study administration

II. Focus group with expert teams

III. Semi-structured interviews with GPs

IV. PTPs and evaluation forms

V. Assessment of DRPs and STOPP and START criteria

VI. Inclusion patient questionnaire

VII. Time registration by expert teams and GPs

VIII. GP EMR data

IX. Patient survey after 3 months

X. Short survey among GPs of control practices

*Rating of implementation fidelity (very low, low, moderate, high)

**Table 2:** Research questions for the evaluation of moderating factors, data sources and outcomes for the implementation fidelity of the Opti-Med intervention

Key intervention components	Data source^1^	Specific research questions	Outcomes
1. Moderating factors: participant responsiveness			
1a. General	I, III, IX	How were patients informed about, and engaged in the intervention? How was the patient recall of consultation? How did patients prepare for the consultation? How did patients perceive the intervention?	Patients received an information letter including a customized leaflet to prepare for the GP consultation GPs found most patients were well informed and pleased with the extra attention for their medication 231 intervention patients filled out the questionnaire at 3 months: 14% of the patients that had a consultation with their GP did not recall the consultation 48% did not prepare particularly for the consultation, 23% brought or studied his/her medication overview, 24% thought of or noted down questions beforehand and 4% brought someone to the consultation 88% of the patients perceived the consultation as pleasant or very pleasant 72% thought the consultation was useful or very useful
2. Moderating factors: strategies to facilitate implementation			
2a. Patient selection	I	What strategies were used to support patient selection?	Patient selection was carried out using a specially designed ICT application that searched GP EMR records on the basis of the study inclusion criteria. Due to difficulties in applying the application and time restraints only a few practice nurses were able to carry out the patient selection independently. The majority needed help from the researchers
2b. Patient involvement	I	What were strategies to support implementation of the intervention and patient involvement?	The patient questionnaire and the customized leaflet to prepare patients for the consultation were strategies to involve patients in their own CMR and tailor it to their needs Patients could ask for assistance in filling out the patient questionnaire on actual medication use and DRPs as needed to prepare the medication analysis. Only a very limited number of patients used this option
2c. Preparation of medication analyses	I	What strategies were used to support the preparation of the medication analysis?	Although time-consuming and prone to error, convenient use was made of the STRIPA. Collecting information from the GP EMR and pharmacy was also convenient but time-consuming due to limitations of the GP IT systems
2d. Medication analysis	I, II	What strategies were used to support expert teams in implementing the medication analyses? How were these strategies perceived by the expert teams?	Expert teams followed an online course, 5 h professional training to prepare for the medication analyses and a 2 h feedback meeting after 2 months into the intervention. During the first sessions all expert teams were assisted by the researchers to help with the software package and available for questions. STRIPA was used to support the medication analyses The training was deemed useful, especially to get acquainted with STRIPA and with the fellow expert team member. Most skills and knowledge were acquired during the course of the study. The expert teams all indicated that the STRIPA was a big support for the structured medication analysis. A barrier was that STRIPA was not supporting the drafting of PTPs
2e. GP consultation	I, III	What were strategies to support implementation of the intervention by the GPs and practice nurses? How were these strategies perceived by the GPs?	Intervention GPs were informed by the researchers during a kick-off meeting and received printed materials on the intervention. Practice nurses received a workbook with practical steps and the researchers assisted the practice nurses when needed and were available for questions via e-mail or phone. We tried to adapt the PTP forms to a format usable in the GP EMR but integration proved impossible to integrate the PTP How the kick-off meeting printed materials and communication was perceived is unknown. GPs indicated that once they got used to the PTP evaluation forms they were easy and structured but some considered the non-compatibility with the GP IT system a barrier
3. Moderating factors: quality of delivery			
3a. Patient selection	II	How was the quality of the patient selection and how was this evaluated by the GPs?	Quality of the patient selection is not relevant and not addressed.
3b. Patient involvement	IV	How was the quality of the patient involvement?	Implementation rate of DRPs modified on basis of patient input was significantly higher as compared to DRPs not modified on basis of patient input (respectively 60 and 46%, *t* test *p* < 0.001)
3c. Preparation of medication analyses	II, III	How was the quality of the preparation of the CMRs and how was this evaluated by the expert team and GPs?	The quality of the preparation was good but occasionally medical or medication files were incomplete. The quality of the medical files differed between GP practices. As a consequence in these cases recommendations were less useful and it required more effort of the GP to conduct the patient consultation. However, GPs reported that in most cases incorrect data could be easily corrected and incorrectly proposed interventions were ignored or adjusted
3d. Medication analysis	III, IV, V	How was the reproducibility of the medication analysis? To what extent were DRPs and proposed interventions related to the STOPP and START criteria in the intervention patients? How was the quality of the medication analyses evaluated by the GPs?	PTP reproducibility between different expert teams was moderate. A mean [sd] of 1.5 [1.2] in the number of DRPs and 2.4 [1.4] deviations in type of DRPs was found per patient between two different expert teams In total 33.1% of the DRPs was related to a STOPP criterion and 19% to a START criterion. For details see Table 3 GPs considered PTPs of good quality and more elaborate than they were used to from other polypharmacy projects or community pharmacist initiatives
3e. GP consultation	IX	How was the quality of the GP consultation according to the patient, in terms of understanding and asking questions?	82% indicated to understand everything or almost everything during the consultation 75% indicated that they could ask all questions or almost all questions during the consultation
4. Moderating factors: context			
4a. General	I, III, X	How did the organization of GP practices affect the implementation? How did attention for polypharmacy in primary care and in society affect the implementation? To what extent was the usual care in the control group implemented as planned? (possible contamination)? How were expert teams and GPs reimbursed?	There were differences between GP practices in how easy the intervention was embedded into daily practice. Implementation went much smoother in GP practices in which a practice nurse was assigned to organize this type of interventions. Personnel changes during the course of the study were barriers for continuation of the intervention and good implementation GPs in the intervention group found specific attention for polypharmacy, medication reviews and the primary care guideline encouraging and considered them important GP care topics The 11 control GP practices confirmed that no structured CMRs were conducted, except in a small student project. However, all practices were involved in an elderly care project with extra attention for pharmacotherapy but only a small number of their patients was involved No reimbursements were offered to patients. Expert teams were paid an hourly rate for their work in the medication analyses. GP practices were paid per patient included

*CMR* clinical medication review, *DRP* drug related problem, *EMR* electronic medical record, *GP* general practitioner, *IT* information technology, *PTP* pharmacotherapeutic treatment plan, *START* screening tool to alert doctors to right treatment, *STOPP* screening tool of older person’s prescriptions, *STRIPA* systematic tool to reduce inappropriate prescribing assistant

^1^I. Study administration

II. Focus group with expert teams

III. Semi-structured interviews with GPs

IV. PTPs and evaluation forms

V. Assessment of DRPs and STOPP and START criteria

VI. Inclusion patient questionnaire

VII. Time registration by expert teams and GPs

VIII. GP EMR data

IX. Patient survey after 3 months

X. Short survey among GPs of control practices

## Data sources

The following data sources were used to address the specific research questions.

### Study administration

Data on selection, inclusion and drop-out of participants, time planning, performing medication analyses by the expert teams, and consultations with the GP were recorded by the researchers alongside the RCT.

### Focus group with experts

A focus group was held with seven members (one GP, two elderly care specialists and four pharmacists) of the four expert teams to collect data on their experiences with conducting the medication analyses. The meeting lasted 70 min and was audio recorded. To facilitate the discussion a topic list was developed beforehand (online resource 1).

### Interviews with the patients’ GPs

From each intervention practice that performed more than ten consultations, a GP was invited for an semi-structured interview; all participated. The interviews were held by the researchers, lasted 15–30 min and were audio-recorded. The objective of the semi-structured interviews was to discuss the experiences of the GPs with this method of conducting CMRs. To facilitate the interview a topic list (Electronic Supplementary Material 3) was developed.

### Evaluation of the implementation of the results of the medication analyses

An evaluation form was used by the GPs to record the follow-up of the changes in the medication regime as proposed by the expert team, including the reason(s) why (part of) these proposals were not implemented. The expert team also indicated for each proposal whether this was influenced by the input of the patient via the questionnaire.

### Classification and assessment of DRPs

The changes in the medication regime as proposed by the expert teams were classified by the researchers (FW, JH) into DRPs using the DOCUMENT DRP classification system [21].

For a random sample of 21 (8%) of all patients a medication analysis was performed by two different expert teams to assess reproducibility.

Subsequently, the STOPP and START criteria were applied to these DRPs to establish their external validity. STOPP (Screening Tool of Older Person’s Prescriptions) is a list of medications that are potentially inappropriate for older people. START (Screening Tool to Alert doctors to Right Treatment) is a list of medications that should be prescribed for older people for a number of conditions. The assessment was carried out by one researcher (HvD) by means of an iterative process. Eventual difficulties were discussed with a second researcher (FW) until consensus was reached. A random sample of 10% of the patients was independently assessed by a second researcher (FW).

### Patient questionnaire

At inclusion, patients completed a questionnaire about their actual medication use and experienced problems with their medication. The patients indicated whether they filled out the questionnaire independently or whether they received help.

### Time registration

The time investment of the expert teams and the GPs in the intervention practices for completing the respective elements of the intervention was calculated by the researchers.

### Electronic medical records

Data on gender and age from the GPs’ EMRs was used for the non-responder analysis.

### Patient survey

The intervention patients completed a survey 3 months after baseline. The survey assessed the preparation and usefulness of the CMR and satisfaction about the consultation with the GP.

### Survey among GPs in control practices

GPs from the control practices received a short survey to assess whether CMRs were conducted unintentionally during the study period for patients of the control group.

## Analyses

Descriptive statistics were used for quantitative data using SPSS Statistics 23, using *t* tests for continuous variables and χ^2^ statistics for categorized variables.

For qualitative analyses, audio files were transcribed verbatim. Transcripts of the focus group and interviews were coded by two independent researchers (respectively FW and MD, and FW and SY) top-down with a pre-defined code-list which was formulated based on the topic lists and knowledge of the intervention. Differences in coding were discussed until consensus was reached, a few codes were added retrospectively. Citations and coded transcripts were arranged to broader themes using Atlas.ti software [22].

## Results

Outcomes per key intervention component for each dimension of the framework are shown in detail in Table 1 and 2.

## Adherence to the intervention

Patient selection was carried out according to the inclusion criteria. However, for this topic, we deviated from the study protocol, most practice nurses did not carry out patient selection and invitation themselves due to difficulties in using the newly-developed software application and due to time restraints. The Opti-Med researchers provided extensive support or carried out the patient completion themselves instead.

Also, the Opti-Med researchers collected most information (GP EMR data, medication overview from pharmacy and patient questionnaire) for the medication analyses instead of the practice nurses, due to time restraints.

Nineteen percent of all DRPs identified were based on the input from the patient questionnaire. The majority of these DRPs were related to medication knowledge or adherence to medication.

The expert teams carried out medication analyses for all but one of the 275 participants of the intervention group and for all 243 control patients. According to the expert team members, medication analyses were conducted in a highly structured manner, mainly due to use of the STRIPA tool. They also mentioned that the method and high number of medication analyses by fixed couples improved efficiency and collaboration. The expert team members and the GPs mentioned the ‘external’ nature of the team as an additional value, because of the fresh perspective of such a team allowing an independent ‘objective’ assessment.

In 90% (247/275) of the patients, GPs discussed the proposed changes in medication with their patients. 42% of the patients had their consultation within the planned first month after inclusion. The method of consultation was deliberately not specified by the researchers. Most GPs planned double consultation time and used a few minutes to prepare the consultations using the PTP.

**Fig. 3:**
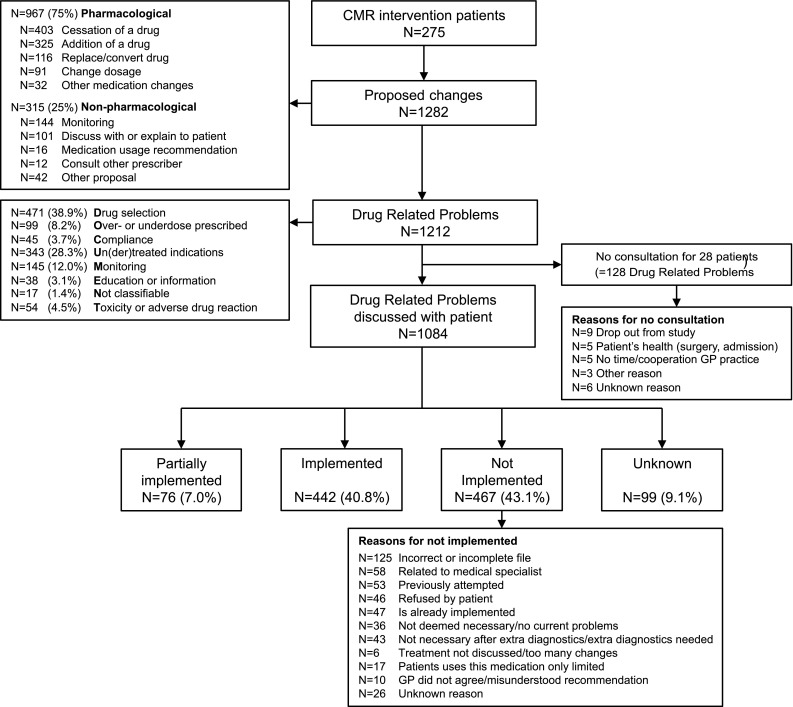
Frequency and nature of proposed changes and drug related problems. For 275 intervention patients, 1282 pharmaceutical and non-pharmaceutical changes were proposed by the external expert teams. Retrospectively, the researchers identified 1212 drug related problems with the DOCUMENT tool [21], out of these proposals

Figure 3 gives an overview of the frequency, nature of DRPs and proposed changes in medication as well as their implementation rate, and reasons for not implementing as proposed. Nearly 50% of all proposed medication changes were (partially) implemented (consented implementation).

‘Addition of a drug’ was significantly more often implemented than ‘cessation of drug’ (46.7 vs. 34.7% (*t* test, *p* = 0.002). The implementation rate of non-pharmacological recommendations (e.g. laboratory tests) was significantly higher than proposed changes in medication (69.2 vs. 42.6% (*t* test *p* < 0.001). The most frequent reasons for non-implementation were: ‘proposed change is based on incomplete medical or medication files’, ‘prescription originates from a medical specialist in secondary care’ or ‘the change in medication has been tried before by patient and/or prescriber’.

The total time spent by all healthcare providers for one patient was estimated at 94 min. This includes 1 min for patient selection, 15 min for preparation, 22 min per expert team member for medication analysis and 34 min for GP consultation.

## Moderating factors

### Participant responsiveness

Over half of the patients reported to have prepared themselves for the consultation with the GP by bringing or studying their own medication, preparing questions, or bringing someone to the consultation. Fourteen percent of the patients who had a consultation with the GP did not recall it. Of the patients who did recall the consultation, the majority considered it useful.

### Strategies to facilitate implementation

Patient selection was facilitated by software specifically developed for the Opti-Med study. However, most practice nurses considered it difficult to use and time consuming. Collecting information from the GPs’ EMRs and pharmacy records in preparation of the medication analyses was useful but time-consuming. The quality of the preparation for the medication analysis was deemed sufficient by the expert teams.

Training in performing CMRs was deemed useful by the expert team members. However, they indicated that most knowledge and skills were acquired when performing the medication analyses. The use of the STRIPA tool was found to greatly support and to highly structure the medication analysis. Some GPs indicated that the form with the PTP was not very user-friendly; however, after a few consultations, most GPs became familiar with it. Seventeen percent of the patients reported to have been assisted in completing the patient questionnaire.

### Quality of delivery

The GPs considered the PTPs drafted by the expert teams of very good quality.

The mean difference between the number of DRPs per patient identified by two expert teams was 1.5 (standard deviation (SD) 1.2) and the mean number of differences in type of DRPs was 2.4 (SD 1.4).

In total 33.1% of the DRPs identified were related to a STOPP criterion and 19% to a START criterion (Table 3), but a considerable part of the identified DRPs could not be related to a STOPP or START criterion (e.g. practical medication problems, changes in dosage or evaluation of drug effect).

**Table 3:** Prevalence of STOPP-START among intervention patients per DOCUMENT DRP type

DOCUMENT DRP type	Total	STOPP	START
	*N* (%)	*N* (%)	*N* (%)
**D**rug selection	471 (38.9)	372 (30.7)	17 (1.4)
**O**ver or underdose prescribed	99 (8.2)	7 (0.6)	3 (0.2)
**C**ompliance	45 (3.7)	3 (0.2)	1 (0.2)
**U**n(der)treated indications	343 (28.3)	1 (0.1)	212 (17.5)
**M**onitoring	145 (12.0)	0	0
**E**ducation or Information	38 (3.1)	0	0
**N**ot classifiable	17 (1.4)	0	0
Toxicity or ADR	54 (4.5)	18 (1.5)	0
Total	1212 (100)	401 (33.1)	233 (19.2)

*ADR* adverse drug reaction, *DRP* drug related problem, *START* screening tool to alert doctors to right treatment, *STOPP* screening tool of older person’s prescriptions

DRPs were identified by the expert team at baseline and classified by the researchers according to the validated DOCUMENT [21] classification system to categorize DRPs into 8 categories. Retrospectively, STOPP and START criteria were assigned to the DRPs

The majority of the patients indicated that they could ask (almost) all questions and understood (almost) everything during the consultation with the GP.

The implementation rate of proposed medication changes influenced by patient input was significantly higher as compared to the implementation rate of proposed changes not influenced by patient input (respectively 60 and 46%, *p* < 0.001).

### Contextual factors

GPs considered the increased attention for polypharmacy, medication reviews, and the recently published Dutch multidisciplinary guideline on polypharmacy [7] encouraging and important for GP care. CMRs were not performed for patients in the control practices, therefore contamination was minimal.

The embedding of the Opti-Med intervention varied between GP practices. GPs and practice nurses reported less complaints and questions from patients when a practice nurse was specifically assigned to the organization of the intervention. GPs mentioned that personnel changes during the course of the study was a barrier for the continuity and implementation of the intervention.

## Discussion

For all key intervention components the implementation fidelity was moderate to high. Almost all key intervention components were generally carried out as planned. However, for the elements patient selection and preparation of the CMR analyses the researchers were more involved than intended. Almost half of the proposed changes in medication were implemented, starting new medications seemed easier than stopping medications. Patient involvement may also be considered accomplished as planned, one-fifth of the proposed medication changes was influenced by patient input.

Training of the expert teams, the use of the STRIPA tool and the structured PTP forms facilitated implementation of the intervention. Difficulties with patient selection due to non user-friendly software and incomplete medical and medication files used for the medication analyses appeared factors promoting non-adherence to the intervention. The reproducibility of the medication analyses between the expert teams was moderate. There were differences in the embedding of the intervention between GP practices. A designated and motivated practice nurse was an important contextual facilitating factor for adherence to the intervention.

To our knowledge, this is one of the first comprehensive process evaluations of a CMR intervention study. Other studies on CMRs did not included or only a limited process evaluation or a different method of CMR [23, 24]. A comparison with previous studies is therefore difficult, however, some results can be compared.

The implementation rate of proposed medication changes of almost 50% is within the range found in other studies [25, 26], higher implementation rates may be found when the patient’s own pharmacist and GP are involved in the medication analysis and less non-relevant recommendations may be formulated. However, GPs did not experience the irrelevant recommendations as inefficient and time consuming and reported that this disadvantage often was outweighed by the advantage of the efficiency, objectivity and expertise of the external expert team.

The 94 min time spent is acceptable compared to other studies and estimations in guidelines [7, 27]. Almost a quarter of the time is spent by the practice nurse instead of the GP and/or pharmacist, which is less costly. However the time investment is still considerable, but may reduce over time. A previous study with Opti-Med data shows that the expert teams can improve the efficiency over time [28.]

The moderate reproducibility of the medication analyses between the expert teams could be partly explained by variations among experts. In a recent Dutch qualitative study on case vignettes with polypharmacy and multimorbidity, it was concluded that GPs varied in medication management strategies which resulted in differences in proposed medication changes [29].

### Lessons learned for CMRs in a non-RCT setting

This process evaluation provides a better insight into the implementation fidelity of an innovative method for CMRs. Implementation fidelity was studied alongside a pragmatic cluster RCT, which does not resemble daily practice. E.g., the efforts and time investment of the researchers are applicable in daily practice.

As the selection of patients and preparation of the CMRs in this study was mainly performed by researchers there are still some barriers to overcome before these key intervention components can be successfully implemented in daily practice. Time, training and dedication of a practice assistant or practice nurse in the GP practice for CMRs are necessary.

The medication analyses being performed by external expert teams seems feasible, however reimbursement and organization of expert teams outside the scope of a research project will be necessary. Currently in The Netherlands GPs and pharmacists are reimbursed for conducting CMRs. A dedicated coordinator may be needed to organise the work of expert teams within e.g. an existing regional collaboration structure between GPs and/or pharmacists.

Reimbursements for the GPs and reminders by the researchers for GPs and patients may have increased the implementation rate of the GP consultations. Of the invited patients, almost 60% did not reply or indicated that they did not want to participate. It might be that in daily practice, a part of this group may need a different approach with possibly more face-to-face contact to identify the actual medication intake, DRPs and preferences.

Identified barriers for implementation in daily practice, such as time restrains and incompleteness of medical files are commonly known from other pharmaceutical care studies or evaluation projects [8, 24, 30].

### Limitations

Several limitations may have influenced the evaluation of the adherence to the intervention and moderating factors determining the implementation fidelity of the intervention.

First, the researchers who carried out the Opti-Med intervention were also involved in the process evaluation. We used a subjective rating to measure implementation fidelity, an objective rating is impossible in this type of process evaluations.

Second, as compared to the framework of Hasson the moderating factors ‘comprehensiveness of the policy description’ and ‘recruitment’ have not been included in the present evaluation. Comprehensiveness of the policy description was not assessed since the number of key components in the intervention is limited and it was not feasible to obtain an external assessment of the policy description with respect to the complex intervention. Recruitment is covered under the adherence dimension ‘coverage’. Furthermore, not all dimensions of adherence and of the moderating factors have been assessed extensively. The assessment of the quality of delivery of the intervention for GP consultations and patient involvement was very limited. Video recordings of consultations might have provided more insight into the quality of delivery. The duration and topic list of the GP interview was limited. Finally, results from a patient survey gave us only limited insight into the patients’ responsiveness and quality of delivery of the patient involvement, compared to e.g. qualitative patient interview data.

## Conclusion

Overall, the implementation fidelity was moderate to high for all key intervention components of the CMR intervention. This means that almost all intervention key components were delivered as intended. The absence of its effectiveness with respect to enhancing quality of life cannot be explained by insufficient implementation fidelity. Nevertheless, this process evaluation provides insight into how this method of conducting CMRs can be implemented in daily practice. Barriers on organizational level must be overcome; the availability of user-friendly software, easy exchange of medical and medication data, and coordination and management of the intervention within a larger collaboration between GPs and pharmacists are very important for successful implementation.

## Electronic supplementary material

Below is the link to the electronic supplementary material.

Supplementary material 1 (DOCX 13 kb)

Supplementary material 2 (PDF 213 kb)

Supplementary material 3 (EPS 1163 kb)
