# Supplementary material for: Implementation fidelity of a clinical medication review intervention: process evaluation
Source: Int J Clin Pharm. 2018 Mar 20;40(3):550–65. doi: 10.1007/s11096-018-0615-y (PMC5984963; doi:10.1007/s11096-018-0615-y)
Supplement: Supplementary file 1 — Supplementary material 1 (DOCX 13 kb) [file 11096_2018_615_MOESM1_ESM.docx]

**Important abbreviations**

CMR=Clinical Medication Review

DOCUMENT=DRP classification system; Drug selection; Over or underdose; Compliance; Un(der)treated indications; Monitoring; Education or information; Not classifiable; Toxicity or ADR

DRP=Drug Related Problems

EMR=Electronic Medical Record

GP=General Practitioner

PTP=Pharmacotherapeutic Treatment Plan

START= Screening Tool to Alert doctors to Right Treatment

STOPP= Screening Tool of Older Person’s Prescriptions

STRIPA=Systematic Tool to Reduce Inappropriate Prescribing Assistant (online tool)
