# Supplementary material for: Implementation fidelity of a clinical medication review intervention: process evaluation
Source: Int J Clin Pharm. 2018 Mar 20;40(3):550–65. doi: 10.1007/s11096-018-0615-y (PMC5984963; doi:10.1007/s11096-018-0615-y)
Supplement: Supplementary file 2 — Supplementary material 2 (PDF 213 kb) [file 11096_2018_615_MOESM2_ESM.pdf]

Electronic supplementary material II

Topic guide semi-structured interviews GP (and practice nurse)

Objective: What are the experiences of the GPs with the Opti-Med medication review proposed interventions and consultations?

<u>I: Evaluation medication review proposed interventions and consultations</u>

- Could you tell me a little about how the Opti-Med intervention was organized and implemented in your GP practice?

- What were your expectations beforehand of medication reviews, especially the feasibility and usefulness for you and the patient?

- How were the consultations with the patients?
  Prompts:
  - *what went good and what went wrong;*
  - *which problems were handled in the consultations;*
  - *were there differences between the first consultations and later on?*

- Were the proposed interventions from the expert teams comprehensible?
  Prompts:
  - *did you always agree with the proposed intervention?*

- What were the most common reasons to not follow up a proposed intervention?

- Could you tell me a little about the completeness of the medical and medication data on which the proposed interventions by the expert teams were based?

- In your opinion, what did you think of the external party, that did not know the patient, reviewed the medication?
  Prompts:
  - *expectations beforehand and opinion now?*
  - *pros and cons of not knowing the patient?*

- Could you tell me a little about the follow-up and monitoring of Opti-Med patients over time?

- Did you learn something about pharmacotherapy for elderly and did you implement these extra knowledge in practice?

- Did you contact medical specialist and/or pharmacists to consult on medication changes in the context of the Opti-Med study.

- Was the Opti-Med intervention indeed innovative for your GP practice?

- Can you mention other elements in the approach and execution of the Opti-Med medication reviews that contributed to high quality of efficient medication reviews or less qualitative or efficient medication reviews?
  Prompts:
    - *point of attention/improvement*
    - *Impact contextual factors (e.g. personnel changes)*

<u>II: Target group</u>

- In your opinion, is the current targeted patient group a useful group for medication reviews?
  Prompts:
  - *interesting/useful subgroups (polypharmacy, oldest old etc?*

- In this study patients with geriatric problems (instability, immobility, incontinence and impaired cognition) were the target group and a new angle for the medication reviews. In your opinion, were these geriatric problems dealt by means of the medication reviews?

III: Implementation in daily practice

- Do you think the Opti-Med method could be implemented in daily GP and pharmacy practice, and how?
  Prompts:
  - *feasibility/priority*
  - *organisation*
  - *role of practice nurses*
  - *cooperation*
  - *training/education*

Topic guide focusgroup discussion expert teams

Objective: What are the experiences of the expert team members with the Opti-Med medication review analyses?

<u>I. Evaluation of the method of performing reviews; facilitators and barriers</u>

- Could you tell me a little about using the structure of the medication analysis (STRIP)?
  Prompts:
  - *did you follow the individual steps of the STRIP guideline and helped that to improve quality and/or efficiency?*
  - *you start with assigning all medications to condition, then undertreatment, overtreatment etc?*

- Did you use the STOPP- and START criteria and/or Dutch GP guidelines (NHG).
  Prompts:
  - *this is mostly embedded in the STRIP-assistant, did you consult guidelines in addition?*

- Could you tell me a little about the knowledge you had before and after the training and the knowledge you derived from performing the medication reviews?
  Prompts:
  - *did you have sufficient knowledge and training to perform the medication reviews?*

- In your opinion, was the data complete provided to perform the medication reviews?
  Prompts:
  - *how often did it occur that essential information was missing to perform a good medication review, or possibly there was too much information?*
  - *in your opinion, were there possibly errors of data entry or in the original files from the GP or pharmacy.*

- How do you feel about the fact that you did not know the patient?
  - *cons and pros*

- What did you think of the cooperation and discussion between physician and pharmacist within the expert team?
  Prompts:
  - *what were you expectations beforehand, and how is this currently?*
  - *was there of was there no consensus?*
  - *complementary knowledge/skills/approach?*

- What did you think of drafting the pharmacotherapeutic treatment plan for the GP?

- What were important differences of Opti-Med compared to regular medication reviews (as far as your experiences reach)
  Prompts:
  - *structure*
  - *time-investment*
  - *quality*

- Can you mention other elements in the approach and execution of the Opti-Med medication reviews that contributed to high quality of efficient medication reviews or less qualitative or efficient medication reviews?
  Prompts:
  - *point of attention/improvement*

II: Target group

- In your opinion, is the current targeted patient group a useful group for medication reviews?
  Prompts:
    - *interesting/useful subgroups (polypharmacy, oldest old etc?*

- To what extent could you take into account the geriatric problems with respect to medication changes or other proposed interventions?

III: Surplus value patient information

- According to you, what is the surplus value of the patient information to tailor the pharmacotherapeutic treatment plan?
  Prompts:
    - *what type of interventions were proposed?*

- Is this input comparable to information which you would derive from a face-to-face contact with the patient?

IV: Implementation in daily practice

- Do you think the Opti-Med method could be implemented in daily GP and pharmacy practice, and how? Especially the use and organization of external expert teams?
  Prompts:
    - *what form could we use for teams?*
    - *who has which responsibility*
    - *role of elderly care specialist*
    - *training/education*

V: Evaluation of STRIP-assistant

- To what extent does the use of the STRIP-assistant, contributed to high quality and efficient medication reviews? Do you have points for improvement for the STRIPA decision-support web application facilitating the pharmacotherapeutic analysis?
